# Breast Implant-Associated Anaplastic Large-Cell Lymphoma: A Case Report

**DOI:** 10.4274/tjh.galenos.2019.2019.0162

**Published:** 2019-11-18

**Authors:** Hakan Kalyon, Erman Öztürk, Sıtkı Tuzlalı, Olga Meltem Akay, Burhan Ferhanoğlu

**Affiliations:** 1 American Hospital, Clinic of Hematology, İstanbul, Turkey; 2 Medeniyet University Faculty of Medicine, Department of Hematology, İstanbul, Turkey; 3 Tuzlalı Pathology Laboratories, İstanbul, Turkey; 4 Koç University Faculty of Medicine, Department of Hematology, İstanbul, Turkey

**Keywords:** Breast implants, Lymphoma, Large-cell, Anaplastic, Seroma

## To the Editor,

Breast implant-associated anaplastic large-cell lymphoma (BIA-ALCL) is a rare type of peripheral T-cell lymphoma, also recognized as a specific disease in the 2016 revision of the World Health Organization classification of tumors of the hematopoietic and lymphoid tissues [1]. Although BIA-ALCL has an indolent course, infiltrative forms may be life-threatening and 9 deaths have been reported [2]. The annual incidence is estimated as 0.1 to 0.3 per 100,000 women with implants [3]. The median age is 53, with the disease being detected after a median of 8 years following implantation [4]. Herein, we report a rare case of BIA-ALCL, the first from Turkey.

**Figure 1:**
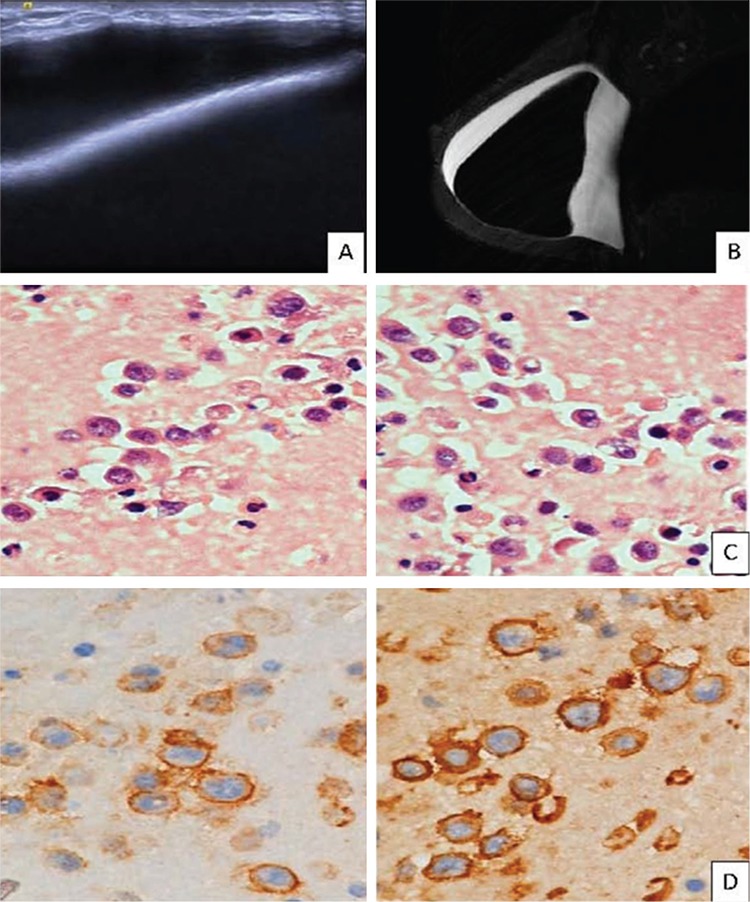
A&B: Ultrasound (A) and magnetic resonance imaging (B) of the capsule of the implant and the seroma at breast. C: Hematoxylin eosin staining, large cells, pleomorphic cells with abundant cytoplasm. D: CD30 (+) lymphocytes.

A 40-year-old Caucasian female presented to our clinic with right-sided breast swelling and asymmetry. Five years ago, she was diagnosed with left-sided invasive ductal carcinoma. She received neoadjuvant chemotherapy, followed by mastectomy and axillary lymph node dissection of the left side and nipple-sparing mastectomy of the right side. Macro-textured anatomical silicone gel implants and fat grafting were applied, followed by adjuvant chemotherapy. Five years later, breast ultrasound and MRI revealed effusion in the fibrous capsule surrounding the breast implant (Figures 1A and 1B). Initial evaluation of the effusion was benign and the implant was replaced by another one after partial capsulectomy. However, the seroma recurred. In the third sampling, the immunochemical analysis revealed typically large and pleomorphic CD30-positive so-called hallmark cells (Figures 1C and 1D). She was diagnosed with BIA-ALCL. The Ann Arbor stage was IE and the TNM stage was IA. Complete excision of the breast implant and capsule was performed and no capsule invasion was reported upon pathological evaluation. Neither further surgery nor chemotherapy was applied. She has remained in remission to date, at the 18^th^ month after the surgery.

Although it is a very rare entity, detection and diagnosis of BIA-ALCL is an emerging topic. BIA-ALCL is surgically treated and it has an indolent course, with the risk of death being 0.4 micromorts per patient [5]. Most cases are unilateral; however, rare bilateral cases have been reported. Patients mainly present with malignant effusions associated with the fibrous capsule surrounding the implants [6]. Lack of ALK expression and strong membranous expression of CD30 constitute the main immunochemical profile. The largest series published in the literature are summarized in Table 1.

The pathogenesis of BI-ALCL is still unclear. Textured implants are likely to induce a marked local T-cell immune response compared to smooth implants. Textured implants are known to shed silicone particulate. Macrophages digesting silicone particulate form foamy cells and release cytokines, eliciting T-cell chemotaxis and replication. These findings help us to hypothesize that BI-ALCL originates from aberrant reactive T-cell populations [7]. The main treatment is surgical removal of the implant and total capsulectomy with complete excision of any associated mass until reaching negative margins on final pathologic evaluation, defined as complete surgical excision. Removal of the contralateral breast implant is controversial, as bilateral capsule involvement was reported in the literature [6,8]. Although there is no randomized controlled trial managing patients with incomplete capsulectomy, with residual disease and with stage II-IV disease, the postulated approach is chemotherapy with cyclophosphamide, doxorubicin, vincristine, and prednisone (CHOP) [6]. CHOP plus etoposide and brentuximab vedotin are alternatives for ALCL treatment [7].

Our patient’s diagnosis was based on CD30 positivity and the presence of large pleomorphic cells. Immunohistochemical staining for ALK was not performed and this is a limitation of our report. Immunohistochemical evaluation of the expressions of CD2, CD3, CD4, CD5, CD7, CD8, CD30, and ALK is necessary and constitutes a widely accepted strategy to evaluate seroma samples.

As the number of breast implant surgeries is rising continuously, the diagnosis of BIA-ALCL is increasing. Patients undergoing breast implantation should be informed of the risk of lymphoma development. Recurring effusions around the capsule may reveal the suspicion of BIA-ALCL. Patients should be treated with surgery-based treatments. Randomized controlled studies are needed to determine standard chemotherapy protocols.

## Figures and Tables

**Table 1:**
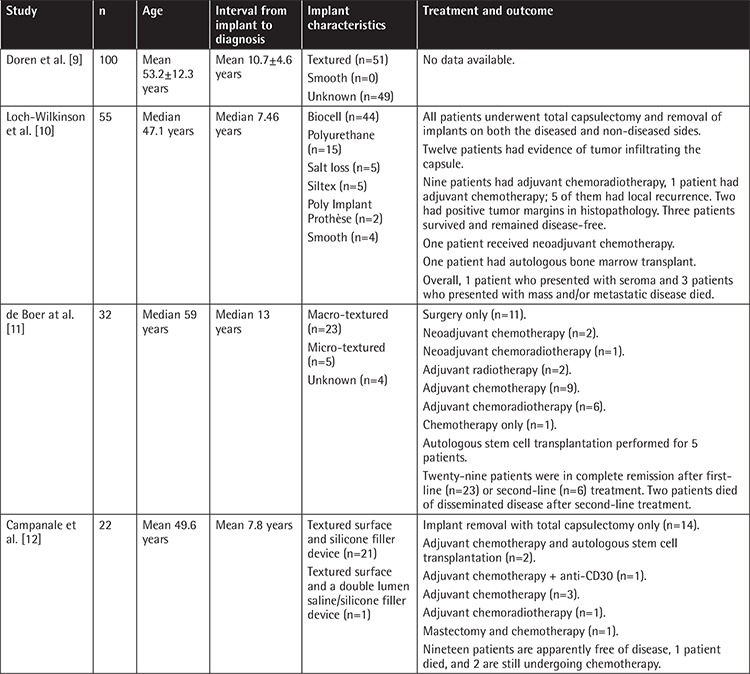
Summary of large series of breast implant-associated anaplastic large-cell lymphoma cases.
